# Deciphering cellular complexity: advances and future directions in single-cell protein analysis

**DOI:** 10.3389/fbioe.2024.1507460

**Published:** 2025-01-14

**Authors:** Qirui Zhao, Shan Li, Leonard Krall, Qianyu Li, Rongyuan Sun, Yuqi Yin, Jingyi Fu, Xu Zhang, Yonghua Wang, Mei Yang

**Affiliations:** ^1^ Yunnan Key Laboratory of Cell Metabolism and Diseases, Yunnan University, Kunming, China; ^2^ State Key Laboratory of Conservation and Utilization of Bio-Resources in Yunnan, Yunnan University, Kunming, China; ^3^ Center for Life Sciences, School of Life Sciences, Yunnan University, Kunming, China

**Keywords:** protein-protein interaction, proteomics, single-cell analysis, single-cell isolation, conventional approaches

## Abstract

Single-cell protein analysis has emerged as a powerful tool for understanding cellular heterogeneity and deciphering the complex mechanisms governing cellular function and fate. This review provides a comprehensive examination of the latest methodologies, including sophisticated cell isolation techniques (Fluorescence-Activated Cell Sorting (FACS), Magnetic-Activated Cell Sorting (MACS), Laser Capture Microdissection (LCM), manual cell picking, and microfluidics) and advanced approaches for protein profiling and protein-protein interaction analysis. The unique strengths, limitations, and opportunities of each method are discussed, along with their contributions to unraveling gene regulatory networks, cellular states, and disease mechanisms. The importance of data analysis and computational methods in extracting meaningful biological insights from the complex data generated by these technologies is also highlighted. By discussing recent progress, technological innovations, and potential future directions, this review emphasizes the critical role of single-cell protein analysis in advancing life science research and its promising applications in precision medicine, biomarker discovery, and targeted therapeutics. Deciphering cellular complexity at the single-cell level holds immense potential for transforming our understanding of biological processes and ultimately improving human health.

## 1 Introduction

Proteins, intracellular and membrane-bound, form the fundamental structural elements of cells and are organized into intricate molecular assemblies facilitating vital cellular processes like transcription, translation, metabolism, growth, adhesion, and signal transduction (Jelokhani-Niaraki, 2022). Consequently, quantifying protein expression levels and analyzing protein-protein interactions (PPIs) are essential for understanding cellular function and regulatory mechanisms. Deciphering these interactions can provide valuable insights into how cells respond to external stimuli, maintain homeostasis, and make fate decisions.

Conventional techniques like Western blotting and RT-PCR quantify collective responses of entire cell populations, assuming that the mean accurately reflects individual cell behavior. However, this assumption overlooks potentially significant variations within subpopulations, which can play pivotal roles in dictating overall population behavior (Hughes et al., 2014; Huang et al., 2023). Cellular processes exhibit inherent heterogeneity, evident in phenomena like stem cell differentiation, development, oncogenesis, immune responses, and neurodegenerative diseases (Mattiazzi Usaj et al., 2021; Liu et al., 2021; Budnik et al., 2018; Ryu et al., 2019a). As well, cells in complex organs, such as the liver, exhibit discrete metabolic and functional roles based on their specific localization (He et al., 2023). The tumor microenvironment (TME) also exemplifies this complexity, with intricate interactions between cancer cells and diverse non-malignant cells, each exhibiting unique behaviors under pathological conditions (Mayer et al., 2023).

To capture cellular diversity, researchers have emphasized developing methodologies for isolating and analyzing individual cells from complex biological samples (Bhagwat et al., 2018; Müller and Nebe-von-Caron, 2010; Pekle et al., 2019; Luan et al., 2020; Grigorev et al., 2023). Single-cell genomic, transcript, and proteomic analyses offer unprecedented insights into genomic variability, gene expression dynamics, and protein expression patterns (Reimegård et al., 2021; Shao et al., 2018). While single-cell RNA sequencing (scRNA-seq) revolutionized our understanding of cell population heterogeneity and gene expression (Reimegård et al., 2021), its limitations, including stochastic expression, mRNA half-life variability, amplification biases, and the inability to capture post-transcriptional modifications, necessitate integrating proteomics for comprehensive understanding (Larsson et al., 2019; Schwanhäusser et al., 2011; Ziegenhain et al., 2017). Protein-protein interactions (PPIs) are fundamental to cellular processes, their presence and strength can vary at the single-cell level. This variability is observed not only across different cell types and tissues but also within populations of genetically identical cells. Measuring PPIs at the single-cell level allows us to capture this heterogeneity and provides insights into how interactions may differ in response to factors like treatment or disease states. For instance, in a study on osimertinib-treated lung cancer models, significant cell-to-cell variation was found in PPIs such as Cyclin E and CDK2, FGFR1 and PIK3R1, and AKT1 and SRC. These interactions can be crucial for understanding how cancer cells respond to treatment and how resistance may develop, emphasizing the importance of single-cell resolution in identifying key interactomic events (Zhang et al., 2024). Single-cell protein analysis, on the other hand, can provide a more direct readout of cellular function, capturing post-translational modifications and protein-protein interactions that are crucial for regulating cellular behavior (Ryu et al., 2019a; Liu L. et al., 2020; Avin et al., 2017).

Recent studies have revealed only modest correlations between mRNA and protein levels (Edfors et al., 2016; Popovic et al., 2018; Liu et al., 2016), underscoring the idea that mRNA expression poorly predicts protein abundance. Proteins exhibit greater stability, and have higher concentrations than mRNAs which minimize random fluctuations. These traits allow proteins to play more immediate roles in sustaining cellular functions compared to transcripts (Liu L. et al., 2020; Liu et al., 2016; Frei et al., 2016). The generally longer half-lives and higher amounts of proteins also make them less susceptible to stochastic variations, allowing for more accurate quantification and analysis at the single-cell level (Popovic et al., 2018).

To comprehensively understand complex cell populations, researchers have developed analytical tools for quantitative and specific single-cell protein detection (Budnik et al., 2018; Ryu et al., 2019a; Bhagwat et al., 2018; Reimegård et al., 2021; Frei et al., 2016; Schulz et al., 2018; Stoeckius et al., 2017; Mimitou et al., 2021; Peterson et al., 2017; Bendall et al., 2012; Lun et al., 2019; Zhu et al., 2019). Operating with or without labels to minimize interference with cellular processes, these tools enable unraveling regulatory circuits, pathways, and mechanisms governing cellular behavior. Single-cell protein analysis has already yielded substantial contributions to our understanding of immune cell heterogeneity, stem cell differentiation, and tumor progression, paving the way for improved diagnostics, targeted therapies, and personalized medicine approaches (Mattiazzi Usaj et al., 2021; Liu et al., 2021; Satija and Shalek, 2014; Giesen et al., 2014).

The advancement of single-cell protein analysis technologies has been accompanied by the development of sophisticated data analysis and computational methods to extract meaningful biological insights from the vast amounts of complex data generated. These methods include data pre-processing, univariate and multivariate analysis, and advanced techniques such as machine learning, which have significantly improved the efficiency and accuracy of single-cell proteomics data analysis (Xie et al., 2020; Liu and Yang, 2021; Liu et al., 2019). As single-cell protein analysis technologies continue to evolve, the development of standardized data analysis pipelines and the integration of multi-omic data will be crucial for obtaining a comprehensive understanding of cellular heterogeneity and function, ultimately advancing our ability to decipher the complexity of biological systems.

This comprehensive review outlines advancements in single-cell separation techniques and single-cell protein analysis. The protein analysis section is further divided into two subtopics: (a) protein expression level analysis and (b) protein-protein interaction analysis. We summarize progress in isolation techniques, including filtration, fluorescence-activated cell sorting (FACS), magnetic-activated cell sorting (MACS), laser capture microdissection (LCM), manual picking, and microfluidics. Furthermore, we discuss breakthroughs in single-cell protein detection and PPI analysis, evaluating system performance in terms of multiplexity, analyte types, throughput, sensitivity, and specificity. We also highlight the importance of data analysis and computational methods in single-cell proteomics and discuss the future directions and potential applications of these technologies in deciphering cellular complexity. While significant progress has been made, challenges related to sensitivity, specificity, and throughput still remain. By comparing advantages and limitations, we provide insights into potential future directions, fostering advancements in this rapidly evolving field and deepening our comprehension of cellular biology and its biomedical applications. Addressing these challenges will be crucial for realizing the full potential of single-cell protein analysis and its widespread adoption in basic and translational research.

### 1.1 Cell isolation

Isolating and accurately identifying target cells is a crucial prerequisite for single-cell analysis. The efficacy of single-cell isolation technologies is predominantly evaluated based on three critical parameters: efficiency, defined as the capacity to isolate a specific number of target cells within a given time frame; separation purity, referring to the proportion of target cells isolated relative to non-target cells post-separation; and recovery rate, indicating the quantity of target cells successfully retrieved post-separation compared to the initial count present in the sample (Hu et al., 2016; Gross et al., 2015). The selection of an appropriate isolation method is essential for reliable single-cell analyses, particularly when studying rare cell populations or investigating cellular heterogeneity, as distinct techniques exhibit varying effectiveness across these parameters.

Contemporary cell separation technologies can be broadly classified into two main categories based on their isolation properties. The first category encompasses techniques that rely on the physical attributes of cells, such as morphology, size, density, and deformability. This category includes methodologies like density gradient centrifugation, which separates cells based on their buoyant density; size-based filtration, which employs membranes with specific pore sizes to isolate cells of interest; manual cell picking, which involves the direct selection of individual cells using micropipettes; and a part of microfluidics-based capture, which utilizes the unique flow properties of cells in microchannels for isolation (Gross et al., 2015; Malter, 2016; Liu and Singh, 2013; Huang et al., 2008; Cha et al., 2022). A key advantage of these techniques is the ability to isolate cells in a label-free manner, without the need for internal or external markers, minimizing potential interference with cellular processes and preserving the native state of the cells (Gross et al., 2015).

The second category relies on the biological properties of cells, employing affinity-based methods. These methods, including fluorescence-activated cell sorting (FACS), magnetic-activated cell sorting (MACS), and laser capture microdissection (LCM), utilize the presence of specific surface proteins and their affinity for corresponding antibodies or probes (Potashnikova et al., 2018; Miltenyi et al., 1990; Vandewoestyne and Deforce, 2010). FACS utilizes fluorescently labeled antibodies to mark cells of interest, enabling their sorting according to fluorescence intensity. MACS uses antibody-coated magnetic beads to separate target cells from a heterogeneous population by use of an external magnetic field. While, LCM employs a laser to selectively isolate cells from tissue sections. These techniques offer high specificity and purity, enabling the isolation of rare cell populations based on their unique biological markers and specific morphology (Gross et al., 2015).

In this section, we briefly summarize the foundational principles, inherent advantages, limitations, and potential applications of the most prevalent cell separation methodologies (Table 1). Each technique’s unique attributes contribute to its suitability for specific research contexts, empathizing the importance of carefully evaluating the requirements of the study and accordingly selecting the most appropriate method. By understanding the strengths and weaknesses of each approach, researchers can optimize their experimental designs and ensure the reliable isolation of target cells for downstream single-cell analysis.

**TABLE 1:** Overview of single cell isolation techniques.

Technology Name	Basic principle	Advantages	Limitations	Applications	Efficiency	Purity
Density gradient centrifugation and Size-based filtration	Leveraging cell morphology, size, density, and deformability	Label-free, easy to operate	May have limited effectiveness for specific cell types	General cell separation needsetc.	Low to Medium	Medium to High
Fluorescence-activated cell sorting (FACS) and Magnetic-activated cell sorting (MACS)	Based on the affinity between cell surface proteins and antibodies	High specificity, yields high-purity cell samples	Costly, requires specific markers	Specific applications requiring high purity, such as research and therapeutic use	High	High
Laser-capture microdissection (LCM)	Precisely isolating target cells from solid tissue samples	High precision, suitable for detailed analysis of specific cells	High equipment cost	Isolation from solid tissue	Low to Medium	High
Manual cell picking/micromanipulation	Manual selection or manipulation of individual cells	High precision, suitable for detailed analysis of specific cells	Low efficiency, labor-intensive	Manipulation of live cells	Low to Medium	High
Micro- and Nanowell Arrays and Droplet Microfluidics	Manipulating liquid at microscale through microfluidics technology to capture and analyze single cells	High-throughput analysis, suitable for large-scale cell analysis and single-cell secretion detection	Complex technology, requires skilled operators	Single-cell isolation, single-cell level biomolecular analysis, single-cell secretion detection	Medium to High	Medium to High

### 1.2 Fluorescence activated cell sorting (FACS)

Fluorescence-Activated Cell Sorting (FACS), an advanced flow cytometry technique with sorting capabilities, and it is widely recognized as the most sophisticated and accessible method for identifying, categorizing, and isolating specific cell types from heterogeneous cell populations (Sharon et al., 2013; An and Chen, 2018; Herzenberg et al., 2002). This versatile technique differentiates cells based on a multitude of parameters, including size, granularity, and fluorescence intensity patterns arising from labeling with specific fluorescent markers. FACS enables concurrent quantitative and qualitative multi-parametric analysis of individual live or fixed cells at impressive throughput rates, rendering it an essential tool for single-cell isolation and characterization.

The FACS process begins with the preparation of a single-cell suspension, wherein target cells are labeled with fluorescent markers. Fluorophore-conjugated monoclonal antibodies, which recognize specific cell surface markers, are the primary choice of fluorescent probes utilized in this technique (Pan and Wan, 2020). As cells traverse through the cytometer’s fluidic system, they are hydrodynamically focused into a single-file stream and individually exposed to one or more laser beams. The interaction of the laser light with the cells generates fluorescence signals, which are collected by dedicated detectors (Adan et al., 2017). The forward scatter (FSC) and side scatter (SSC) signals provide information about the cell’s relative size and granularity, respectively, while the fluorescence detectors identify and characterize cells based on their predefined fluorescence signatures (Adan et al., 2017).

**FIGURE 1:**
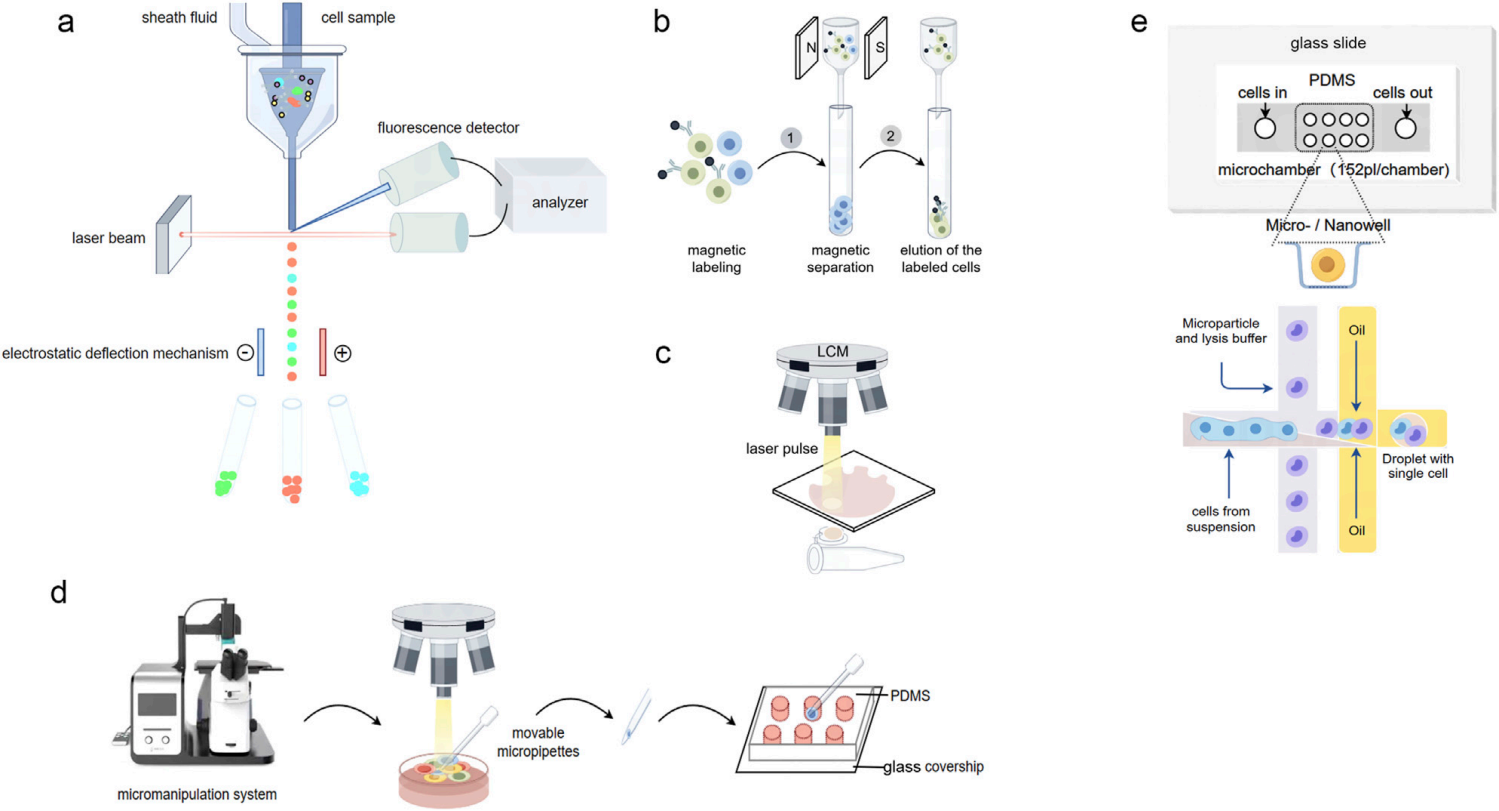
Overview of single-cell isolation technologies. **(A)** Schematic of fluorescence-activated cell sorting. As cells coated with fluorescent antibodies traverse through the cytometer, they are individually illuminated by a laser beam, enabling fluorescence detectors to identify and sort cells based on predefined characteristics. **(B)** Schematic of magnetic-activated cell sorting. When a heterogeneous cell mixture is exposed to an external magnetic field, desired cells labeled with magnetic beads become magnetically responsive. **(C)** Schematic of laser capture microdissection. The technique utilizes a laser to cut the tissue and let the cells adhere to the melted membrane. **(D)** Schematic of manual cell picking. The cells of interest are retrieved under a microscope by glass capillary transferred to microwells for further analysis. **(E)** Schematic of microfluidic used for single cell isolation. Upper: microwell Arrays for Single-Cell isolation; Lower: discrete aqueous droplets formation in droplet microfluidics.

The sorting mechanism of FACS relies on an electrostatic deflection system. After laser excitation, the cell stream is fragmented into individual droplets, with each droplet encapsulating a single cell. Droplets can be formed by using high-frequency (cycles/second, Hz) vibration of the nozzle at an optimal amplitude. Based on the detected fluorescence and scatter signals, the droplets containing the desired single cells are assigned an electrical charge—either positive or negative by an electrical charging ring placed just at the point where the stream breaks into droplets. Next, as the droplets pass through an electric field, the charged droplets are deflected into designated collection tubes or well plates for downstream analysis or culture, while the uncharged droplets are directed into a waste container (Figure 1A) (Rambault et al., 2021). State-of-the-art FACS systems, such as the FACS-Aria™ III, boast an impressive throughput rate of up to 100,000 droplets per second, enabling the analysis of approximately 70,000 events per second (Gross et al., 2015). Moreover, FACS has proven adept at sorting individual cells from complex mixtures containing thousands of cells, utilizing up to 18 distinct surface markers for precise identification and separation (Hu et al., 2016). Additionally, an advanced FACS system developed by Dr. Li’s group has demonstrated over 90% recovery efficiency and more than 80% cell viability (Dong et al., 2017).

One of the key advantages of FACS over other cell sorting techniques is its capability to analyze and sort cells simultaneously based on multiple parameters, encompassing both surface and intracellular markers (Bennett et al., 2016). This multi-parametric capability enables highly specific cell isolation and characterization, allowing researchers to identify and isolate rare cell populations with unique phenotypic profiles (Maes et al., 2020).

After single cell isolation, subsequent analysis is necessary. The high throughput and purity of FACS make it an invaluable tool in various applications, including immunophenotyping (An and Chen, 2018; Pan and Wan, 2020), cell cycle analysis (Potashnikova et al., 2018), subpopulation analysis (Antoniadi et al., 2022), cancer diagnosis (Sharon et al., 2013), and the isolation of rare cell populations for downstream single-cell analyses, such as single-cell RNA sequencing and single-cell proteomics (Zhu et al., 2019).

Despite its unparalleled versatility, FACS has certain limitations. The technique requires a relatively large initial cell number, typically in the range of 10 ^ 4 to 10 ^ 6 cells, which can hinder the isolation of individual cells from rare subpopulations (Gross et al., 2015; Shields et al., 2015). Moreover, the high-speed fluidic system and the potential for shear stress during sorting may compromise the viability and functional integrity of sorted cells, particularly for fragile cell types (Gross et al., 2015). To mitigate these issues, researchers have developed gentle sorting techniques, such as low-pressure sorting and the use of cell-friendly collection buffers, to improve cell viability and recovery (Teteris et al., 2012). Furthermore, recent advancements in microfluidics-based cell sorting technologies have enabled the isolation of single cells from rare populations with minimal sample requirements and reduced shear stress (Stavrakis et al., 2019).

### 1.3 Magnetic-activated cell sorting (MACS)

Magnetic-Activated Cell Sorting (MACS) is another widely employed technique for the passive separation of cell populations, leveraging their cluster of specific extracellular markers to isolate specific cell types (Rambault et al., 2021; Shen et al., 2021). MACS has demonstrated the capability to achieve isolation of particular cell groups with a purity exceeding 90%, ideally even higher than 95% (Miltenyi et al., 1990; Desikan et al., 2022) and high recovery rate of more than 90% (Willasch et al., 2010). The principle underlying MACS involves the use of magnetic beads conjugated with targeting molecules such as antibodies, enzymes, lectins, or streptavidin. These conjugated beads are designed to selectively target surface molecules primarily on live cells, thereby enabling the efficient isolation of the desired cell population.

MACS offers two modes of separation: positive and negative. In positive separation, the heterogeneous cell mixture is incubated with magnetic beads coated with antibodies specific to the surface markers of the target cells (Pan and Wan, 2020). When exposed to an external magnetic field, the labeled cells become magnetically responsive and are retained within the field, while unlabeled cells can be easily removed through a washing process (Figure 1B). This approach is particularly useful when the target cell population is rare or when a high purity of the isolated cells is required (Nemescu et al., 2020). In contrast, negative separation is employed when targeting cell-specific substances are unavailable or when the aim is to deplete a specific cell population from the sample. In this mode, the unwanted cells are labeled with more than one antibody-conjugated magnetic beads and subsequently separated from the mixture, thereby isolating the unlabeled target cells in the solution (Pan and Wan, 2020).

Compared to FACS, which can separate cells based on the expression of multiple specific molecules simultaneously, MACS has a more limited capacity, sorting cells solely into positive and negative populations based on a single surface marker. Consequently, the purity level achievable with MACS is typically lower than that of FACS (Zeb et al., 2019). However, MACS offers several advantages, including its relative simplicity, cost-effectiveness, and the ability to process large sample volumes quickly. Additionally, MACS is generally gentler on the cells than FACS and results in lower cell loss, as it does not require high-pressure fluidic systems, making it more suitable for isolating fragile cell types (Sutermaster and Darling, 2019). To enhance the performance of MACS, an advanced method known as integrated Dielectrophoretic-Magnetic Activated Cell Sorter (iDMACS) was developed in 2009 (Kim and Soh, 2009). This innovative approach combines dielectrophoretic forces and magnetic particles. In iDMACS, cells are first labeled with magnetic beads and then subjected to a dielectrophoretic force, which helps to further separate the labeled and unlabeled cells based on their distinct dielectric properties. This additional separation step improves the purity of the isolated cell population (Kim and Soh, 2009). Recently, a novel purification method of human iPSC-derived cells at large scale by combining the miR-switch and MACS (miR-switch-MACS) was developed in 2022. This method achieved over 99% purity of chromogranin A-positive cells following puromycin selection to eliminate untransfected cells, surpassing the commonly used 95% purity threshold (Tsujisaka et al., 2022).

MACS enables efficient enrichment of target cell populations based on their unique surface markers, rendering it a valuable tool for a wide range of biological and medical research applications where high-purity cell samples are indispensable (Nemescu et al., 2020; Li et al., 2023). Its ability to process large sample volumes rapidly and its relative simplicity make it an attractive choice for many cell isolation tasks. Moreover, MACS can be used in conjunction with other cell separation techniques, such as FACS or density gradient centrifugation, to achieve even higher purity and specificity (Berteli et al., 2017). As the field of single-cell analysis advances, the innovation of new magnetic separation technologies and the fusion of MACS with microfluidic platforms show potential in applications of this robust cell isolation technique (Wang X. et al., 2019). Wang et al., combined Microfluidic chip with magnetic-activated cell sorting technology. Compared with EpCAM dependent traditional CTCs isolation system like CytoQuest™ CR system, their antigen-independent platform maintained a high detection sensitivity while achieving higher CTC counts consistent with clinical observations.

### 1.4 Laser capture microdissection (LCM)

Laser Capture Microdissection (LCM) is an advanced technology for the precise isolation of pure cell populations or individual cells from solid tissue samples mounted on a microscope slide, as established by Emmert-Buck et al. (1996). This technique enables the targeted capture of cells of interest from heterogeneous tissue sections, allowing for subsequent molecular analyses such as PCR (Kandathil et al., 2013), microarrays (Wang et al., 2010), and proteomics (Dilillo et al., 2017).

The procedure for LCM begins with the visualization of target cells under a microscope. The tissue sample is typically prepared as a thin section and mounted on a special microscope slide. The operator then identifies the cells of interest and marks the section to be excised on the display by outlining it with a line. Subsequently, a laser follows this path to cut the tissue, isolating the desired cell or compartment (Figure 1C). The laser cutting procedure remains consistent across various LCM systems, but there are different methods for retrieving the dissected tissue, including contact-based extraction (Vandewoestyne et al., 2013), gravity-assisted microdissection (Gross et al., 2015), and laser pressure catapulting (Vogel et al., 2007). Contact-based extraction involves utilizing a thermoplastic film placed in contact with the tissue section. When activated by the laser, the film melts onto the targeted cells, enabling their separation from the surrounding tissue. In gravity-assisted microdissection, the laser cuts the tissue, and the dissected cells fall into a collection tube due to gravity. Conversely, laser pressure catapulting employs a focused laser pulse to propel the cut tissue into a collection device positioned above the sample. Typically, fixed cells—most commonly used in LCM—are subjected to a single downstream analysis. However, different downstream applications require distinct recovery protocols (such as variations in lysis solvents, extraction reagents, and treatment conditions) to obtain DNA, RNA, proteins, or metabolites (Guo et al., 2023).

Laser Capture Microdissection (LCM) offers several significant advantages. One of the most notable is its ability to maintain both precision and versatility when working with both fixed and live samples (Espina et al., 2007). LCM enables the accurate separation of even small numbers of cells, as well as single-cell isolation with high purity. With recent advancements in optical resolution, LCM can now also isolate cell organelles (Satori et al., 2012). This feature allows researchers to study cells in their native tissue environment, preserving the spatial relationships and potential interactions between different cell types. Additionally, LCM minimizes damage to adjacent cells following the initial dissection, ensuring that the collected cells are not contaminated by unwanted material. LSM, when combined with other techniques, has become more widely used in single-cell protein analysis. Gordon and Gousset demonstrated the integration of Laser Capture Microdissection (LCM) with mass spectrometry (MS) to identify low-abundance proteins in complex samples, achieving high protein extraction efficiency and excellent sample quality (Gordon and Gousset, 2021). However, LCM has some limitations, including a high operational barrier due to the need for specialized equipment and trained personnel, relatively low throughput compared to other cell isolation methods, and potential UV-induced damage to DNA or RNA during operation (Espina et al., 2007).

To overcome these limitations, recent advancements in LCM technology have focused on improving the speed, automation, and gentleness of the cell capture process. For example, the development of infrared (IR) LCM systems has enabled the use of longer wavelengths of light, reducing the risk of UV-induced damage to biomolecules (Vandewoestyne et al., 2013). Moreover, the integration of LCM with other technologies, such as microfluidics and single-cell sequencing, has expanded its applications and enhanced its throughput (Zhang et al., 2022).

Integration with immunohistological staining enhances LCM as a robust tool for analyzing solid samples at the single-cell level. By employing specific antibodies to mark cells of interest, researchers can visualize and isolate these specific cell types based on their protein expression profiles. This capability renders LCM an invaluable tool for diverse research efforts that necessitate the isolation of specific cells for subsequent molecular analysis, such as studies on tumor heterogeneity, neurodegenerative disorders, and developmental biology (Vandewoestyne and Deforce, 2010; Fink et al., 2006; Decarlo et al., 2011).

### 1.5 Manual cell picking/micromanipulation

Manual cell picking, also known as micromanipulation, represents a straightforward, convenient, and effective approach for isolating single cells. Micromanipulation systems typically involve the use of an inverted microscope in conjunction with movable ultrathin glass capillaries controlled by motorized micromanipulation stages (Figure 1D). The cell sample is usually suspended in a dish or well-plate, allowing for direct visualization and precise isolation of individual live or fixed cells. The operator can observe and photograph the cells under the microscope, facilitating isolation based on morphological characteristics or fluorescent labeling.

A primary advantage of manual cell picking is its capability to isolate cells in a live state, preserving their viability and functionality for subsequent applications like single-cell sequencing, clonal expansion, or functional assays (Hu et al., 2016). This feature sets it apart from techniques like LCM, which primarily isolate single cells from sections of fixed tissue. Manual cell picking enables researchers to select and extract specific cells of interest based on their morphology, behavior, or response to stimuli, providing a powerful tool for studying cellular heterogeneity and elucidating the roles of individual cells within a population (Malter, 2016; Fröhlich and König, 2000; Li et al., 2011).

### 1.6 Microfluidics

Microfluidics is a rapidly growing field of science and engineering that focuses on the manipulation and control of fluids at the microscale level. This technology enables the development of highly miniaturized platforms, often referred to as “lab-on-a-chip” devices, capable of handling biological samples in extremely small volumes, typically in the nanoliter range. The miniaturization offered by microfluidics provides several advantages in the study of biological systems, particularly in the realm of single-cell analysis and manipulation (Grigorev et al., 2023; Sims and Allbritton, 2007; Lecault et al., 2012; Liu D. et al., 2022).

Microfluidic systems offer a significant advantage in generating localized high protein concentrations. By confining cells and their secreted proteins within small volumes, requirement for amplification techniques to detect low-abundance proteins is not that necessary with microfluidic devices, enabling more precise quantification of biomolecules derived from single cells (Liu and Singh, 2013). This feature is particularly valuable in the study of rare cell types, such as hematopoietic stem cells and circulating tumor cells (CTCs), where sample availability is often limited (Yao et al., 2014). Furthermore, the integration of microfluidic cell isolation and detection strategies enables extensive parallelization, allowing for the quantitative analysis of hundreds to thousands of single cells simultaneously (Yu et al., 2014).

Microfluidic technologies encompass a diverse array of principles and techniques for single-cell isolation (Figure 1E). It is suitable for both live and fixed cells, with a recovery rate exceeding 90% and a purity greater than 90% for single-cell isolation. However, these values may vary depending on the experimental conditions (Yeo et al., 2016; Liu et al., 2018; Wang K. et al., 2019; Pritchard et al., 2019). Two of the most prominent approaches are microwell arrays and droplet microfluidics.

#### 1.6.1 Microwell arrays for single-cell isolation

Microwell-shaped microfluidic devices are widely used for single-cell capture. These devices feature arrays of thousands of nanoliter-scale compartments that effectively trap individual cells, enabling high-throughput analysis and subsequent detection of cellular or secreted biomolecular signals within the confined microwells (Zhao et al., 2023). The detection of these signals can be achieved through surface-modified glass coverslips or nanobeads. The optimization of single-cell purity can be achieved by adjusting the microwell size according to the given sample. These systems can simultaneously process a large number of cells in parallel, making them suitable for applications such as studying antibody specificity, isotype, and affinity secreted from thousands of B cells (Story et al., 2008). While microwells offer the advantage of straightforward operation and fabrication, one potential limitation is the uneven distribution of reagents. To address this, several efforts have been made to achieve a more uniform flow field distribution (Chen et al., 2024; Hu et al., 2018).

#### 1.6.2 Droplet microfluidics for single-cell isolation

Droplet microfluidics, introduced in the early 2000s, has emerged as a promising technique for the isolation of single cells (Ou et al., 2021). This approach utilizes oil-filled channels to generate and contain discrete aqueous droplets, enabling efficient cell isolation. In droplet generation, Gallium electrodes integrated within a microfluidic chip is commonly used to release a droplet as required with high specific spatial or temporal resolution (Berlanda et al., 2021). Femtoliter droplets of different viscous solutions could be produced by deforming an aqueous–oil interface inside a microchannel using a pulsed electric field (Shojaeian et al., 2019). Another approach employed two Laplace pressure barriers to generate droplets by first filling a reservoir before pinching off droplets into a main channel (Totlani et al., 2020). After droplet generation, fluorescence-activated droplet sorting is a well-established technique and can be found in many microfluidic devices. Besides, passive and active droplet sorting methods have also advanced in recent years. For example, Pan et al. showed that the interfacial tension changes with the pH, when specific surfactants are chosen, which allows for passive and label-free sorting based on pH (Pan et al., 2019). Passive high-throughput size-based sorting of hydrogel droplets was realized by inertial forces resulting in cross-streamline migration (Li et al., 2018).

The use of microfluidic droplets offers several notable advantages. First, it significantly reduces sample and reagent consumption, as the droplet volume typically ranges from femtoliter to nanoliters (Jiang et al., 2023). Secondly, the monodisperse nature and large spacing between microdroplets minimize cross-contamination, which is particularly important when working with cells. Thirdly, this technique allows for high throughput, enabling the isolation of several thousand single cells per second (Edd et al., 2008). Additionally, droplet microfluidics provides precise control and high reproducibility. These droplets also serve as convenient micro reaction compartments for subsequent analysis.

After single cell isolation, microfluidics has found wide application in cell analysis, such as small molecule detection (Sun et al., 2019), protease activity analysis (Stucki et al., 2021), gene function studies (van Tatenhove-Pel et al., 2020), and more (Table 2). For small molecule detection, Raman scattering (SERS)-microfluidic droplet platform is commonly used. Xu’s group applied this platform to enable the label-free simultaneous analysis of multiplexed metabolites, like pyruvate, adenosine triphosphate and lactate, at the single-cell level (Sun et al., 2019). Microfluidic droplet technology has broad applicational prospects in single-cell protein analysis, especially for secreted proteins, because of its relatively small size. Dynamic detection and analysis of specific proteins in single cells is still challenging by conventional flow cytometry. Yu et al. reported a microfluidic approach for the detection of MMP9 enzyme activity in individual tumor cell droplets using a flow-focusing capillary microfluidic device (Wu et al., 2021). Wimmers et al. presented a microfluidic single-cell droplet system for the immunofluorescence detection of type I interferon (IFN) production in human plasmacytoid dendritic cells (pDCs) (Wimmers et al., 2018). Its ability to handle large numbers of single cells in a highly controlled and reproducible manner has rendered it an indispensable technique for high-throughput single-cell manipulation and analysis in the field of biomedicine.

**TABLE 2:** Applications of microfluidic studies.

Application	Key finding	Reference
Single cell secreted exosomes	They developed a platform to profile five phenotypic exosomes from over 1,000 single cells simultaneously.	Song et al. (2022)
Single-cell proteomics	Combining chip-based sample handling with DIA-MS using project-specific mass spectral libraries, they profile on average ∼1,500 protein groups across 20 single mammalian cells.	Gebreyesus et al. (2022)
Single-cell isolation	They use thermal bubble micropump technology to drive the fluid flow, and single-cell isolation is achieved by matching the flow resistance of the flow channel.	Xu et al. (2023)
Intracellular Proteins in Single Cells	They developed a microfluidic method to profile protein expression in individual cells by performing single-cell intracellular protein immunoassay in picoliter paired droplets.	Liu et al. (2022b)
purification of lung cancer cells	They developed a spiral microfluidic device that can rapidly isolate cancer cells and improve their purity through fluid dynamics.	Tsou et al. (2020)

In summary, microfluidics provides a robust tool for the precise manipulation, analysis, and investigation of cellular heterogeneity at the individual cell level. The miniaturization, high throughput capacity, and precise control provided by microfluidic technologies empower the generation of single-cell data, facilitating the acquisition of valuable biological insights into diverse cellular systems (Satija and Shalek, 2014; Zhao et al., 2014). As the field of microfluidics progresses and merges with other single-cell analysis techniques, it is poised to play a crucial role in furthering our comprehension of cellular heterogeneity and its implications in diverse biological processes.

## 2 Measurement of protein expression level at single cell resolution

### 2.1 Fluorescence or image-based measurements of endogenous protein levels

Endogenous gene tagging with fluorescent proteins or fluorophore-conjugated antibody labeling techniques have enabled highly sensitive quantification of endogenous protein levels. The fluorescence intensity obtained from these methods directly correlates with the quantity of protein molecules present, thereby enabling the assessment of relative protein abundance.

Fluorescence labeling of target proteins combined with FACS is considered the gold-standard approach for profiling proteins at the single-cell level (Hu et al., 2016). This method involves staining single cells with fluorescent-labeled antibodies or tagging them with a fluorescent protein, which are then detected as they pass through the flow chamber during FACS analysis. The fluorophores are excited by a laser, and the emitted fluorescence is subsequently measured. Through meticulous system calibration using protein-coated beads, the fluorescent intensities can be translated into single-cell protein expression levels (Adan et al., 2017).

FACS facilitates high-throughput detection (∼10^4^ cells/s) and simultaneous measurement of ∼20 multiplexing membrane and intracellular protein parameters (Liu L. et al., 2020). However, this technique has some limitations. Dynamic cell monitoring over time poses a challenge, and the multiplexing capacity is constrained by spectral overlap, potentially impacting the accuracy of protein measurements. In the multi-colour detection system, each excitation laser, including green (532 nm), blue (488 nm) and violet (407 nm) ranges and red (633 nm), is configured with multiple detectors (for the green laser, fluorochromes PE, TRPE, Cy5PE, Cy5.5PE and Cy7PE could be applied and separated). If additional fluorochromes become available that have distinct emission spectra after excitation by the green laser, the detection capacity could be increased even further. Furthermore, physical stressors experienced by cells during FACS procedures could influence their status. A critical aspect is the requirement for a large number of cells (1 × 10^6/mL) in conventional flow cytometry sample preparation (Xie and Ding, 2022).

To address these challenges, flow cytometers incorporating microfluidics have been proposed as a solution (Liu et al., 2011; Wu et al., 2012; Su et al., 2011). Wu et al. introduced a microfluidic platform that integrates cell preparation and multi-color flow cytometry components. This on-chip system offers multiplexed and orthogonal data for profiling signaling pathways across various cell types, including primary cells (Wu et al., 2012). The integration of microfluidics with flow cytometry enables the analysis of smaller sample sizes, reduces the risk of sample contamination, and allows for the precise control of the cellular microenvironment during analysis. Besides, a recent study showed a workflow for generating double emulsions and performing multicolor cell sorting using a commercial FACS instrument. This workflow achieves a double emulsion detection rate exceeding 90%, enabling multicellular encapsulation and high-throughput immune cell activation sorting for the first time (Ding et al., 2024).

Despite these challenges, FACS remains a powerful tool for single-cell protein analysis, offering high throughput and multiplexing capabilities. Researchers continue to explore and develop new techniques that address these limitations, aiming to enable more comprehensive and accurate analysis of protein dynamics and interactions at the single-cell level.

### 2.2 Single-cell Western blotting (scWB)

**FIGURE 2:**
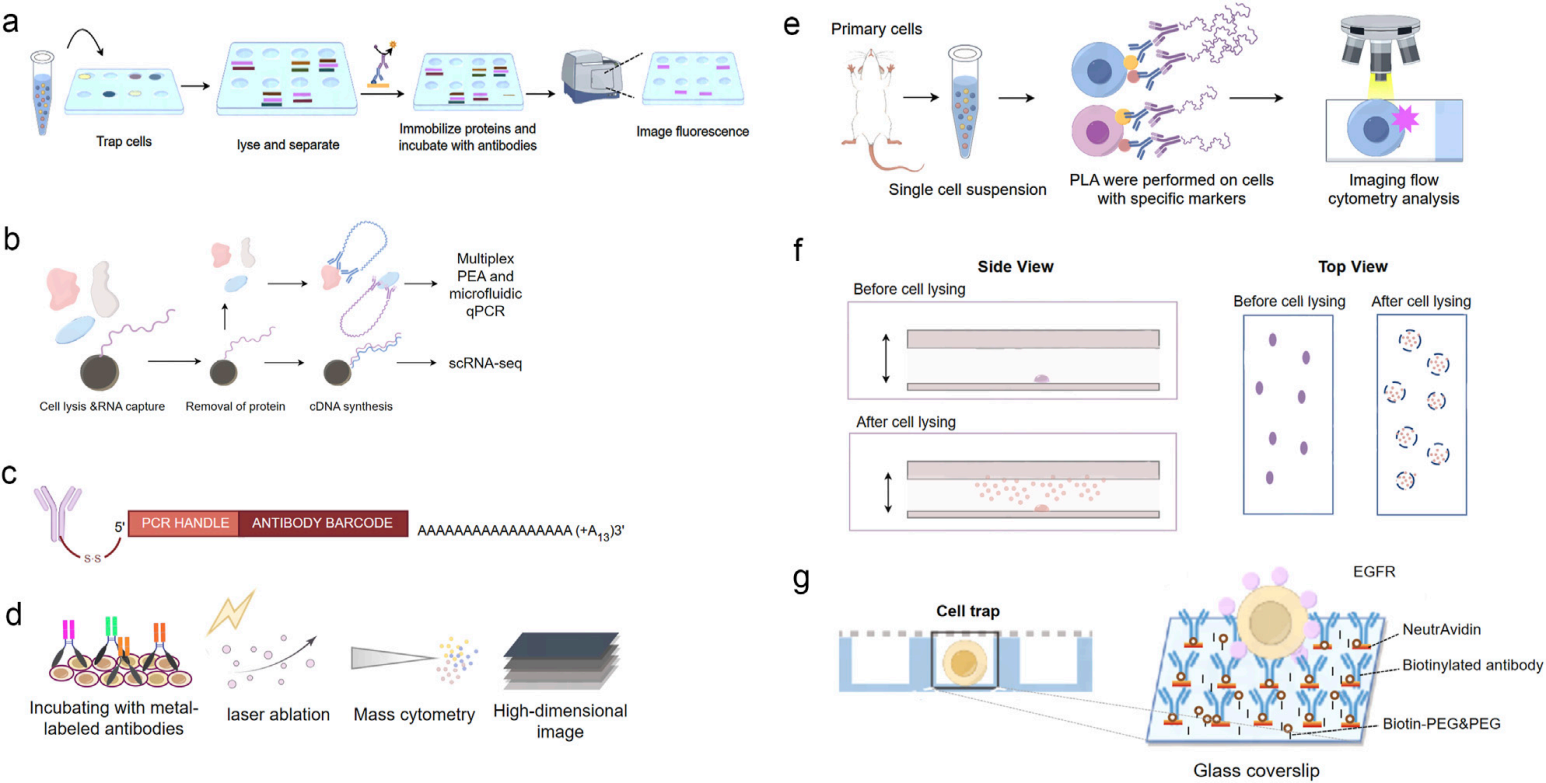
Overview of single-cell protein analysis technologies. **(A)** Schematic of Single Cell Western blotting. Single cells are placed onto the microwells and lysed *in situ*. The proteins from the lysed cells are then separated by gel electrophoresis within each microwell. Following electrophoresis, the proteins are immobilized onto the PA gel through photoinitiated blotting. Finally, fluorescent-labeled antibodies are used for protein detection within each microwell. **(B)** Schematic of Single-Cell Protein And RNA Co-profiling (SPARC). Single cells are isolated and lysed. Following oligo-dT mRNA hybridization, the protein-containing supernatant is removed for subsequent multiplex proximity extension analysis (PEA) and the mRNA is processed using a modified Smart-seq2 approach. **(C)** Illustration of the DNA-barcoded antibodies used in CITE-seq. **(D)** Schematic of Mass Cytometry. After incubation with metal-labeled oligonucleotides for RNA detection and antibodies for protein detection, tissues are subjected to laser ablation and mass-cytometric measurement of the metal abundances. The metal abundances per laser shot (in pixels) are then assembled into a high-dimension image. **(E)** Schematic of the Proximity Ligation Imaging Cytometry assay. **(F)** Schematics of *in situ* protein capture from single cells. Cells are sparsely immobilized on the coverslip surface. The cells are subsequently lysed, and the proteins released are captured by the antibody surface, where single-molecule imaging is performed. **(G)** Schematic of the single-cell capture trap.

Single-cell Western blotting (scWB) is another innovative technique that combines microfluidics and conventional Western blotting to analyze protein expression at a single-cell resolution (Hughes et al., 2014). It overcomes the issue of cross-reactivity by introducing a step of electrophoretic separation before antibody probing (Figure 2A). The scWestern analysis utilizes a microscope slide coated with a thin photoactive polyacrylamide gel. This gel is micropatterned with an array of thousands of microwells, allowing for the simultaneous analysis of 10^3^–10^4^ single cells/chip. Simply stated, the scWestern approach integrates all the essential steps of Western blotting into a dense array format. In this technique, single cells are placed into the microwells and lysed *in situ*. The proteins from the lysed cells are then separated by gel electrophoresis within each microwell. Following electrophoresis, the proteins are immobilized onto the PA gel through photoinitiated blotting. Finally, fluorescent-labeled antibodies are used for protein detection within each microwell.

scWB has emerged as a promising tool for investigating protein expression in individual cells, offering valuable insights into cellular heterogeneity and dynamics at a higher resolution (Rosàs-Canyelles et al., 2020; Kim et al., 2021; Grist et al., 2020). scWB enables detection of more than 10 proteins, including both membrane and intracellular proteins, in each cell during 4 h. The sensitivity of the scWB method is comparable to that of flow cytometry (FC). However, scWB encounters challenges in quantifying secreted proteins, low-abundance proteins, and small molecular weight proteins (Duncombe et al., 2016). To address these limitations, several new methods have been suggested and developed. For example, the integration of scWB with ultrasensitive detection methods, such as single-molecule fluorescence microscopy or mass spectrometry, has the potential to improve the sensitivity and dynamic range of protein measurements (Yen et al., 2014; Lomeli et al., 2021).

### 2.3 DNA reporter-based measurements of endogenous protein levels

In addition to using fluorescent antibodies for protein expression measurement, employing DNA amplification and reporters offers improved accuracy and higher sensitivity. With protein signal outputs encoded by various DNA sequences, the multiplexing capacity is virtually unlimited when utilizing DNA reporters. Several approaches have emerged to enable simultaneous measurement of both mRNA and protein at the single-cell level (Reimegård et al., 2021; Stoeckius et al., 2017; Peterson et al., 2017; Mimitou et al., 2019).

One such approach is Single-Cell Protein and RNA Co-profiling (SPARC) (Reimegård et al., 2021), proposed by Dr. Gallant’s group in 2021. SPARC enables the simultaneous measurement of overall mRNA levels and targeted 89 intracellular proteins in individual cells (Figure 2B). Single cells were sorted on a BD FACS ARIAIII into a 96-well plate and lysed for protein analysis. Protein quantification is accomplished through multiplex, homogeneous protein extension assay (PEA), which is an affinity-based detection technique employing pairs of antibodies linked with oligonucleotides (Reimegård et al., 2021). In PEA, each antibody pair is designed to recognize a specific protein target, and when both antibodies bind to the same protein, the oligonucleotides are brought into close proximity, allowing for their extension and amplification. The resulting DNA amplicons are then quantified using next-generation sequencing or qPCR, providing a digital readout of protein abundance (Darmanis et al., 2016).

CITE-seq, pioneered by Stoeckius et al., is another well-established method in single-cell analysis (Stoeckius et al., 2017). This innovative technique utilizes a digital, sequencing-based platform to quantify single-cell protein expression levels. It involves conjugating antibodies to oligonucleotides, incorporating a unique barcode for antibody identification and facilitating PCR amplification (Figure 2C). The antibody-oligo complexes are then applied to single-cell suspensions. After incubation, a washing step removes unbound antibodies before proceeding to single-cell RNA sequencing (scRNA-seq). CITE-seq primarily allows for the analysis of cell surface proteins, providing valuable information about cell type, state, and function. Recently, Liu et al., extended co-indexing of transcriptomes and epitopes (CITE) to the spatial dimension and demonstrated high-plex protein and whole transcriptome co-mapping to measure 273 proteins and transcriptome in human tissues with 10–50 μm Spatial resolution (Liu et al., 2023). The simultaneous measurement of mRNA and surface proteins enables a more comprehensive characterization of cellular heterogeneity and can reveal novel cell subpopulations and their functional properties.

The integration of DNA reporters with single-cell protein analysis has greatly expanded the multiplexing capacity and sensitivity of these techniques. The ability to encode protein signals with unique DNA sequences allows for the simultaneous measurement of hundreds to thousands of proteins in individual cells, overcoming the limitations of spectral overlap encountered in fluorescence-based methods (Table 3). Furthermore, the amplification of DNA reporters through PCR or sequencing enables the detection of low-abundance proteins that may be missed by other techniques (Reimegård et al., 2021).

**TABLE 3:** Applications of dna reporter-based measurement of protein level insingle cells.

Application	Key findings	Reference
Spatial CITE-seq	They extended co-indexing of transcriptomes and epitopes (CITE) to the spatial dimension.	Liu et al. (2023)
Identification of senescent cell subpopulations by CITE‐seq	Based on the presence of proteins on the cell surface by using single‐cell CITE‐seq, they identified two distinct IR‐induced senescent cell populations.	Abdelmohsen et al. (2024)
Dynamic gene expression networks in B cell development and transformation	They coupled the surface marker on B-cells by using CITE-Seq with single-cell RNAseq to identify differentially expressed gene networks across B cell development.	Lee et al. (2021)
ASAP-seq	ASAP-seq is a unique approach that enables the concomitant detection of protein abundance alongside transposase-accessible chromatin and mtDNA in thousands of single cells.	Mimitou et al. (2021)
DBiT-seq	They presented deterministic barcoding in tissue for spatial omics sequencing (DBiT-seq) for co-mapping of mRNAs and proteins in a formaldehyde-fixed tissue slide via next-generation sequencing (NGS).	Liu et al. (2020b)

### 2.4 Chemiluminescence and visible color-based measurements of endogenous protein levels

Chemiluminescence (CL) is known for its simple and cost-effective optical systems, as it does not require an external light source. This characteristic circumvents issues related to stray light and the instability of light sources, ultimately resulting in low backgrounds and high sensitivity.

The Enzyme-linked immunospot assay (ELISPOT) is a powerful technique developed in the 1980s for quantitatively detecting individual cells that secrete a specific protein of interest (Alexander et al., 2013). This method, based on an immuno-sandwich assay, is valuable for enumerating cell subsets with distinct secretory functions and for monitoring cellular responses to external stimuli or drugs (DiPiazza et al., 2016; Barabas et al., 2017). In ELISPOT, cells are cultured on a surface coated with antibodies that target the protein of interest. The secreted protein is then captured by the immobilized antibodies. A second, enzyme-conjugated antibody is then added, which binds to the captured protein. Upon addition of a substrate, the enzyme catalyzes a reaction that produces a visible spot on the membrane, with each spot representing an individual secreting cell.

The ELISPOT assay is well-known for its high sensitivity in detecting secreted proteins, with a detection limit of approximately six spots per 100,000 cells (Moodie et al., 2010). It enables high-throughput detection (∼10^6^ cells/run) and simultaneous measurement of 1-3 secreted proteins. It has been particularly useful in studying immune responses, such as the detection of cytokine-secreting T cells or antibody-secreting B cells, and in assessing the impact of various interventions on cell function (Leehan and Koelsch, 2015). The ability to quantify the frequency of secreting cells and the relative amount of protein secreted by each cell makes ELISPOT a valuable tool for understanding the heterogeneity of cellular responses.

Recent advancements in ELISPOT technology have focused on improving its multiplexing capabilities and compatibility with other single-cell analysis techniques. For instance, FluoroSpot, a type of fluorescent ELISPOT, allows for the concurrent detection of multiple secreted proteins by utilizing fluorescent dyes instead of enzymes for signal generation (Axelsson, 2022). Additionally, the integration of ELISPOT with microfluidic devices has allowed for the isolation and analysis of individual secreting cells, providing a more comprehensive profile of their functional properties (Huang et al., 2012).

### 2.5 Mass spectrometry-based measurements of endogenous protein levels

Recent advances in mass spectrometry (MS) have made single-cell MS one of the most powerful approaches to obtain the protein profile of a single cell (Slavov, 2021; Petelski et al., 2021). Mass spectrometry-based methods can generally be divided into two main categories: targeted and untargeted approaches.

#### 2.5.1 For targeted proteins

Targeted MS approaches involve the specific detection and quantification of pre-selected proteins or peptides within a sample. This method is useful when researchers are interested in analyzing a particular set of proteins or biomarkers within a single cell.

Mass Cytometry, also known as CyTOF (Cytometry by Time-of-Flight), represents an innovative fusion of flow cytometry (FC) and mass spectrometry (MS), offering an alternative approach for the identification and quantification of target proteins (Table 4). Applying this technology to tissues or cells on slides, termed imaging mass cytometry (IMC), allows for visualization of normal and diseased tissues *in situ* (Tracey et al., 2021). This method facilitates high-dimensional, single-cell analysis of cell type and state (Giesen et al., 2014). In principle, mass cytometry closely resembles flow cytometry, where cells are labeled with antibodies conjugated with metal isotopes and subsequently analyzed via mass spectrometry. The use of specific isotopes circumvents issues related to spectral overlap, enabling simultaneous, multiplexed detection of target proteins (Zhang et al., 2020).

**TABLE 4:** Applications of cytof studies.

Application	Key findings	Reference
Chronic myeloid leukemia	Combination of BCR-ABL1 inhibition and activation of TP53 promotes anti-leukemic activity and inhibits leukemic stem cells	Carter et al. (2020)
Characterization of the Multiple Myeloma Immune Microenvironment	Patients with multiple myeloma showed CD4^+^ T/CD8+ T ratios decrease along with cancer development by CyTOF.	Yao et al. (2022)
Single-Cell Mapping of Human Brain Cancer	The leukocyte landscape of brain tumors was mapped using high-dimensional single-cell profiling.	Friebel et al. (2020)
Immune cellular profiling	CyTOF analysis of peripheral blood mononuclear cells showed a significantly higher percentage of Th17 cells in active tuberculosis.	Kim et al. (2023)
Hypothalamic circuitry	CyTOF revealed extensive changes to immune cell distribution and functional responses in peripheral blood during hyperarousal.	Li et al. (2020)

The workflow of mass cytometry includes the following steps: (1) cells are labeled with antibodies attached to distinct metal isotopes; (2) these labeled cells are introduced into the mass cytometer, where they are vaporized and ionized; (3) the metal ions are separated according to their mass-to-charge ratio; and (4) the quantity of each metal ion is measured, indicating the expression level of the associated protein (Olsen et al., 2019).

Mass cytometry has been broadly adopted and applied to many biological problems (Schulz et al., 2018; Lun et al., 2019; Krishnaswamy et al., 2014; Ajami et al., 2018). Friebel et al. successfully applied this technique, designed two CyTOF panels together, measuring 74 parameters at the single-cell level (Friebel et al., 2020) (Figure 2D). This innovative approach expands the capacity for comprehensive protein analysis within tissues, providing a valuable tool for studying complex biological systems (insert).

In terms of throughput, mass cytometry allows for the measurement of approximately ∼1,000 cells per second, which is inferior to the throughput achievable with fluorescent-labeled analysis approaches (Liu L. et al., 2020). Additionally, when compared to quantum-efficient fluorophores, mass reporters demonstrate lower sensitivities, posing challenges in the accurate measurement of low-abundance proteins in single cells. However, the high dimensionality and minimal spectral overlap of mass cytometry make it a powerful tool for dissecting the complexity of cellular systems and identifying rare cell subpopulations (Levine et al., 2021).

#### 2.5.2 For untargeted proteins

Recent advancements in liquid chromatography-mass spectrometry (LC-MS)-based proteomics methods have addressed significant challenges related to sensitivity and throughput, making single-cell mass spectrometry (scMS) more feasible. This approach has become increasingly utilized to gain valuable insights into cellular heterogeneity (Budnik et al., 2018; Zhu et al., 2019; Lombard-Banek et al., 2019; Cheung et al., 2021; Schoof et al., 2021). One of the main advantages of scMS is its ability to provide an unbiased, global view of the cellular proteome. Unlike targeted approaches that rely on antibodies, scMS can detect and quantify thousands of proteins without prior knowledge of their identity or function. This untargeted nature of scMS enables the discovery of novel proteins and pathways that may be missed by other methods (Lombard-Banek et al., 2019).

Current scMS approaches can quantify approximately 1,000 proteins per cell and analyze over a hundred cells per day of instrument time (Schoof et al., 2021). Despite having a lower throughput than some other single-cell protein analysis methods, the comprehensive coverage and capability to detect post-translational modifications make scMS a valuable tool for understanding cellular heterogeneity at the proteome level (Liu L. et al., 2020).

## 3 Techniques for analyzing protein-protein interaction at single cell resolution

### 3.1 Proximity-based assay

The Proximity Ligation Assay (PLA) represents a relatively novel method for studying protein-protein interactions (PPIs) with high specificity, applicable in both cells and tissue sections (Söderberg et al., 2006). In the assay, a pair of oligonucleotide-labeled antibodies is used to recognize two target proteins. When the two proteins are in close proximity (less than 30–40 nm apart) due to their interaction, the oligonucleotides are ligated, subsequently extended, and amplified to generate sequence products. Detection is accomplished by adding complementary fluorescently labeled oligonucleotides or by measuring the output of DNA sequencing using qPCR.

Dr. Abramson’s group has integrated the PLA assay with imaging flow cytometry (IFC) and developed Proximity Ligation Imaging Cytometry (FLIC) (Avin et al., 2017). This integration enables multiparametric fluorescent and localization analysis of cellular events, including the examination of PPIs and post-translational modifications (PTMs), as well as the assessment of subcellular signal distribution within cells (Figure 2E). FLIC combines the specificity of PLA with the high-throughput and multiplexing capabilities of IFC, allowing for the simultaneous analysis of multiple PPIs and cellular parameters in thousands of individual cells. Vistain et al. developed Prox-seq, a method that enables the detection of intracellular and membrane proteins, protein complexes, and mRNAs in thousands of single cells. Prox-seq combines single-cell RNA sequencing (scRNA-seq) with the proximity ligation assay (PLA). It utilizes pairs of DNA-conjugated antibodies that, when brought into close proximity, facilitate the ligation of DNA oligonucleotides (oligomers) on the antibodies. This ligation generates a PLA product that can be detected through next-generation sequencing. The method allows for the simultaneous measurement of up to 741 protein complexes across 8,700 single peripheral blood mononuclear cells (Vistain et al., 2022).

To investigate PPIs directly with high spatiotemporal resolution, genetically encoded biosensors based on Förster Resonance Energy Transfer (FRET) or Bioluminescence Resonance Energy Transfer (BRET) can be employed. These resonance energy transfer (RET) techniques offer the ability to record single-cell kinetics with millisecond resolution, facilitating the identification of protein complexes and the study of cell-to-cell heterogeneity (van Unen et al., 2016). Recently, Hoshino et al. designed γB2-FRET probes by fusing FRET donor and acceptor fluorescent proteins to a single γB2 molecule, successfully visualizing γB2 homophilic interactions in cultured hippocampal neurons (Hoshino et al., 2023). By utilizing these biosensors, researchers can visualize dynamic changes in PPIs in real-time, providing valuable insights into the molecular mechanisms underlying various cellular processes.

### 3.2 Co-immunoprecipitation (co-IP) based assays

The Single-molecule pull-down (SiMPull) technique provides a method to directly capture native protein complexes from cell lysates, facilitating the identification of complex composition and PPIs at the single-molecule level (Jain et al., 2012). SiMPull involves immobilizing an antibody against a target protein on a glass surface, followed by the application of cell lysates containing the protein complexes. The captured complexes are then visualized using fluorescently labeled antibodies against the interacting partners, allowing for the direct observation of PPIs and the determination of their stoichiometry.

Expanding on this concept, various techniques have been suggested to investigate PPIs at the single-cell level (Ryu et al., 2019a; Zhao et al., 2023; Wang et al., 2018; Wedeking et al., 2015; Stolpner and Dickinson, 2022). Dr. Ha’s group developed an assay that involves lysing bacterial cells *in situ* and capturing the released proteins on an imaging surface coated with antibodies. This approach allows for the unambiguous assignment of captured proteins to their originating cells (Wang et al., 2018). The developed platform is compatible with high-throughput protein analysis and PPI analysis at the single-cell level through single-molecule imaging (Figure 2F). The ability to link protein interactions to specific cells enables the investigation of cell-to-cell variability in complex formation and function, providing insights into the mechanisms underlying cellular heterogeneity.

For mammalian cells, micro-patterned surfaces have been utilized to capture target cells and proteins. Yoon et al. implemented a method that involved *in situ* co-IP for individual cancer cells (Ryu et al., 2019a). This process included capturing single cells using a microfluidic device and inducing *in situ* lysis of the cells while immunoprecipitating EGFRs (the proteins of interest) to the surface. Subsequently, fluorescently labeled downstream interactors of EGFRs were directly added to stimulate protein-protein interactions, which were then visualized using a single-molecule fluorescence microscope. This innovative approach offers insights into the dynamics of protein interactions at the single-cell level, providing valuable information on signaling pathways and heterogeneity in cancer cells (Figure 2G).

The integration of co-IP with microfluidic devices and single-molecule imaging has greatly enhanced the sensitivity and resolution of PPI analysis at the single-cell level. By capturing protein complexes directly from individual cells, these techniques overcome the limitations of bulk co-IP experiments, which average out the heterogeneity in protein interactions across a population of cells. Moreover, the ability to visualize individual protein complexes allows for the quantitative analysis of their composition, stoichiometry, and dynamics, providing a more detailed understanding of their functional roles in cellular processes.

## 4 Data analysis

The advent of advanced single-cell protein analysis techniques, such as flow cytometry and mass spectrometry (MS), has revolutionized our ability to study cellular heterogeneity and protein dynamics at the individual cell level. These methods generate vast amounts of complex data, necessitating the development of sophisticated data analysis and computational approaches to extract meaningful biological insights.

In flow cytometry, data pre-processing typically involves gating, which entails selecting cells of interest by plotting the data in univariate histograms or bivariate (density) plots and defining an area of interest (Aghaeepour et al., 2013; Malek et al., 2015; Montante and Brinkman, 2019). While manual gating is convenient, it can be time-consuming, subjective, and prone to bias. To address these limitations, numerous semi-automated and automated gating methods have been developed, along with machine learning algorithms such as RchyOptimyx (Aghaeepour et al., 2012) and FloReMi (Van Gassen et al., 2016) that utilize these gating methods to identify cell types exhibiting significant differences between groups or for survival analysis. Following gating, data compensation and transformation are crucial steps to ensure accurate analysis (Tinnevelt et al., 2021). Modeling the distribution of single cells is typically performed using clustering (Aghaeepour et al., 2013), principal component analysis (PCA) (Ma et al., 2017), or t-distributed stochastic neighbor embedding (t-SNE) (Amir el et al., 2013).

Single-cell Mass Spectrometry (MS) stands as another potent technique for studying single-cell profiles due to its high sensitivity, broad detection range, and molecular identification capabilities. However, the raw data acquired from single-cell MS experiments are large and complex, necessitating systematic data analysis approaches (Liu and Yang, 2021). Unlike conventional MS proteomics, data analysis methods in current single-cell proteomics studies lack standardization. There is no widely agreed-upon, consistent analysis pipeline across different researchers and laboratories. The typical workflow for single-cell proteomics data analysis includes data pre-processing, univariate analysis, multivariate analysis, and advanced data analysis techniques (Liu and Yang, 2021). Data pre-processing involves noise and background signal removal, ion intensity normalization (Yin et al., 2018), and selection of proteins commonly detected in the majority of single cells (Pluskal et al., 2010). Univariate analysis, such as t-tests and analysis of variance (ANOVA), is used to reveal changes in cellular proteins corresponding to specific biological processes. Multivariate analysis, including unsupervised methods like PCA (Fang et al., 2020) and t-SNE (Wang R. et al., 2019), and supervised methods such as partial least squares discriminant analysis (PLS-DA) (Sun and Yang, 2019) and orthogonal projections to latent structures discriminant analysis (OPLS-DA) (Walker et al., 2013), are employed to reduce data dimensionality and identify patterns in protein expression.

Recent advancements in machine learning (ML) have introduced new data analysis methods for single-cell identification, proteomics, demonstrating high classification accuracy in distinguishing cell types (Xie et al., 2020), special omics, and enabling the extraction of trace-level signals from high-resolution mass spectra (Liu et al., 2019). Image-Based cell identification can bring significant insight to biomedical sciences. Deep learning algorithms could enable cell classification and isolation based on human-vision uninterpretable features within a complex cell population without labeling (Tang et al., 2023). Machine learning workflow developed by Xie et al., was trained to classify single cells according to their mass spectra based on cell groups of interest (GOI). The trained models achieved >80% classification accuracy (Xie et al., 2020). MEISTER, a mass spectrometry (MS) framework, integrates deep-learning-based reconstruction, three-dimensional (3D) molecular distributions and cell-specific mass spectra to 3D brain-wide single-cell biochemical mapping (Xie et al., 2024).

## 5 Applications and future directions

One of the key applications of single-cell protein analysis is the study of cellular heterogeneity in various contexts. For example, mass cytometry has been used to explore immune cell heterogeneity (Newell et al., 2012), while single-cell Western blotting (Sinkala et al., 2017), microfluidic single-cell imaging (Ryu et al., 2019b), and mass cytometry (Levine et al., 2015) have been employed to investigate cancer cell heterogeneity. These studies have revealed the presence of distinct cell subpopulations and provided insights into cell-cell interactions in complex biological samples, such as breast cancer tissues (Giesen et al., 2014).

In addition to studying cellular heterogeneity, single-cell proteomic technologies have proven to be valuable tools for investigating intracellular protein-protein interactions (PPIs) and signaling networks. By profiling a large number of proteins in individual cells, researchers can conduct pairwise protein expression correlation analysis to study protein activating and inhibitory interactions. Techniques such as microfluidic single-cell single-molecule imaging (Wedeking et al., 2015), proximity ligation assay (PLA) (Avin et al., 2017), and FRET assays (van Unen et al., 2016) have been successfully applied to elucidate PPIs and signaling pathways at the single-cell level.

Despite the remarkable progress made in single-cell protein analysis, there are still challenges to overcome in order to fully understand the complex and dynamic nature of the single-cell proteome. To address these challenges, the development of next-generation single-cell analytical techniques is crucial across multiple areas. One key area of focus is the enhancement of detection sensitivity, which will enable the identification and quantification of low-abundance and less stable proteins. Advancements in droplet microfluidic diagnostic techniques, such as the integration of the “picoliter single-cell reaction flask” principle, have shown promise in screening single cells for secreted molecules, including antibodies, cytokines, enzymes, and metabolites (Lan et al., 2023). Another important direction is the investigation of subcellular compartments and the precise analysis of proteins corresponding to specific organelles. Improved single-cell Western blotting methods, such as subcellular Western blotting techniques, have demonstrated the ability to separately assay proteins in the cytoplasm and nucleus of individual cells (Yamauchi and Herr, 2017).

To achieve a comprehensive understanding of single-cell states and functions, the development of complementary methods that integrate multiple types of data is essential. For example, combining high-throughput techniques for the simultaneous detection of mRNA and intracellular proteins can provide a more complete picture of the molecular regulatory networks within individual cells (Reimegård et al., 2021). Flow cytometry-based FISH (Flow-FISH) has been used to simultaneously measure transcript levels and protein expression in single cells, offering deeper insights into the regulation of gene transcription and translation (Van Hoof et al., 2014).

The application of machine learning (ML) techniques to single-cell protein analysis has the potential to revolutionize data analysis and interpretation. ML methods have significantly improved the efficiency of single-cell proteomics data analysis, enabling the identification of patterns and relationships that may be difficult to detect manually (Xie et al., 2020). As ML algorithms continue to evolve and become more sophisticated, their broader application in single-cell protein analysis is expected to facilitate the discovery of novel biomarkers, therapeutic targets, and disease mechanisms.

While the commercialization and clinical translation of single-cell protein analysis technologies have begun, there are still significant barriers to their widespread adoption in clinical and industrial settings. These technologies often require extensive expertise and hands-on time, and their repeatability may not yet meet the stringent requirements for industrial applications. To overcome these challenges, the development of more user-friendly and robust systems is essential. This can be achieved through device automation and further optimization, enabling researchers and practitioners to implement these methods across a wide range of applications.

## 6 Discussion

The rapid development of single-cell protein analysis has revolutionized our ability to study the protein composition, expression levels, and functions of individual cells (Liu et al., 2021; Ryu et al., 2019a; Reimegård et al., 2021; Shao et al., 2018; Peterson et al., 2017; Maes et al., 2020; Sims and Allbritton, 2007; Xie and Ding, 2022; Pereira et al., 2022; Lohani et al., 2023; Yu et al., 2022; Hoover et al., 2023; Mund et al., 2022). By analyzing membrane proteins, cellular proteins, and secreted proteins, researchers can gain a more comprehensive understanding of cell states, functional changes, and cellular heterogeneity (Budnik et al., 2018; Giesen et al., 2014; Sharon et al., 2013; Rosàs-Canyelles et al., 2020; Crouch et al., 2024). This review provides a comprehensive summary of the advancements in single-cell protein analysis technologies over the past two decades, discussing various methods and techniques, and comparing their approaches for single-cell isolation based on factors such as purity, throughput, and efficiency.

Despite recent developments, current single-cell protein analysis techniques still have limitations that need to be addressed (Liu L. et al., 2020; Hu et al., 2016; Bennett et al., 2023). Improvements are needed in accuracy, reproducibility, and the ability to detect low-abundance proteins (Hughes et al., 2014; Hu et al., 2016; Gross et al., 2015; Lugli et al., 2017). The limited multiplexing capacity of existing methods hinders the comprehensive proteomic detection of over 10,000 proteins within a single cell. Additionally, most techniques cannot simultaneously detect membrane-bound, cytoplasmic, and secreted proteins, restricting our understanding of cell protein composition. Furthermore, current methods for analyzing PPIs are semi-quantitative and limited to known protein pairs, hindering the exploration of novel protein interactions.

The complexity and dynamic nature of proteins present a significant challenge in achieving high throughput, accuracy, multiplexing, and sensitivity simultaneously in single-cell proteomic analysis. Researchers must balance the need for detecting low abundance proteins with the desire to analyze a large number of cells efficiently. Future advancements in technology and methodology are expected to address these challenges, enhancing the capabilities of single-cell proteomic analysis and enabling more comprehensive insights into cellular heterogeneity and function. The integration of single-cell protein analysis with other omics technologies, such as single-cell transcriptomics and metabolomics, will provide a more holistic view of cellular processes and uncover novel regulatory mechanisms (Reimegård et al., 2021; Frei et al., 2016; Stoeckius et al., 2017; Peterson et al., 2017). The application of machine learning techniques to single-cell protein analysis will revolutionize data analysis and interpretation, facilitating the discovery of novel biomarkers, therapeutic targets, and disease mechanisms.

With ongoing technological advancements, single-cell protein analysis is expected to have significant applications in clinical diagnostics, cancer therapy, and drug development. Studying protein expression and PPIs in individual tumor cells can improve our understanding of tumor heterogeneity and drug resistance mechanisms, providing a basis for personalized therapy. Single-cell protein analysis can also aid in screening novel drug targets and assessing the impact of drugs on cell function, accelerating the drug development process (Barabas et al., 2017; Heath et al., 2016; Fitzgerald and Leonard, 2017). As technology advances, collaboration among researchers from different disciplines will be crucial to drive further progress in single-cell proteomics, ultimately leading to a deeper understanding of cellular heterogeneity and its implications for human health and disease.
